# QuickStats

**Published:** 2013-09-27

**Authors:** Ji-Eun Kim, Mary Ann Bush

**Figure:**
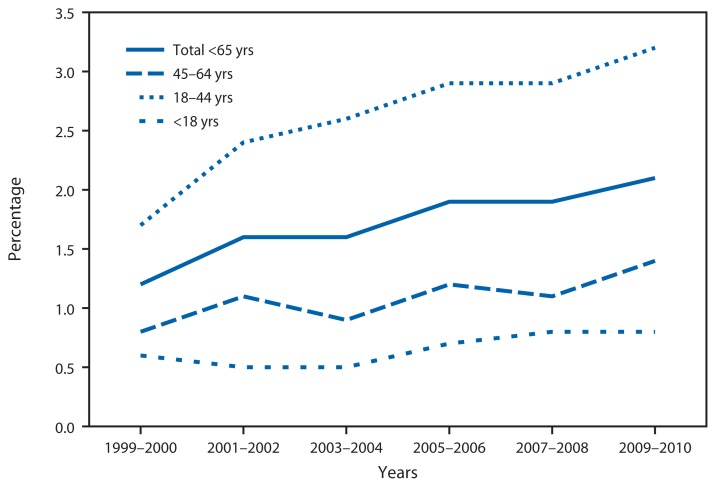
Percentage of Emergency Department (ED) Visits That Were Dental-Related* Among Persons Aged <65 Years, by Age Group — National Hospital Ambulatory Care Survey, 1999–2000 to 2009–2010 * Defined as having a first-listed diagnosis code of 520.00–528.00 in the International Classification of Diseases, Ninth Revision, Clinical Modification.

During 1999–2000, 1.0 million visits to the ED for dental-related problems were made by persons aged <65 years. Dental-related ED visits increased to 2.3 million during 2009–2010, representing 2.1% of all ED visits among those aged <65 years, compared with 1.2% during 1999–2000. Over the same period, the percentage of ED visits for dental-related problems among adults aged 18–44 years increased from 1.7% to 3.2%. Although the percentage of ED visits that were dental-related increased among all age groups aged <65 years during this period, the percentage was higher among adults aged 18–44 years for all years.

**Source:** National Hospital Ambulatory Care Survey. Available at http://www.cdc.gov/nchs/ahcd.htm.

